# Eosinophilic Esophagitis in Children and Adolescents with Abdominal Pain: Comparison with EoE-Dysphagia and Functional Abdominal Pain

**DOI:** 10.1155/2016/4123692

**Published:** 2016-08-16

**Authors:** Thirumazhisai Gunasekaran, Gautham Prabhakar, Alan Schwartz, Kiranmai Gorla, Sandeep Gupta, James Berman

**Affiliations:** ^1^Advocate Children's Hospital, University of Illinois and Loyola Medical Center, 1775 Dempster Street, Park Ridge, IL 60068, USA; ^2^Advocate Children's Hospital, 1775 Dempster Street, Park Ridge, IL 60068, USA; ^3^University of Illinois, Chicago, IL 60607, USA; ^4^University of Indiana, Indianapolis, IN 46202, USA; ^5^Advocate Children's Hospital and Loyola Medical Center, 1775 Dempster Street, Park Ridge, IL 60068, USA

## Abstract

*Aim*. Compare EoE-AP with EoE-D for clinical, endoscopy (EGD), histology and outcomes and also with FAP-N.* Method*. Symptoms, physical findings, EGD, histology, symptom scores, and treatments were recorded for the three groups. Cluster analysis was done.* Results*. Dysphagia and abdominal pain were different in numbers but not statistically significant between EoE-AP and EoE-D. EGD, linear furrows, white exudates were more in the EoE-D and both combined were significant (*p* < 0.05). EoE-D, peak and mean eosinophils (*p*  0.06) and eosinophilic micro abscesses (*p*  0.001) were higher.* Follow-Up*. Based on single symptom, EoE-AP had 30% (*p*  0.25) improvement, EoE-D 86% (*p* < 0.001) and similar with composite score (*p*  0.57 and <0.001, resp.). Patients who had follow-up, EGD: 42.8% with EoE-AP and 77.8% with EoE-D, showed single symptom improvement and the eosinophil count fell from 38.5/34.6 (peak and mean) to 31.2/30.4 (*p*  0.70) and from 43.6/40.8 to 25.2/22.8 (*p* < 0.001), respectively. FAP-N patients had similar symptom improvement like EoE-D.* Cluster Analysis*. EoE-AP and FAP-N were similar in clinical features and response to treatment, but EoE-D was distinctly different from EoE-AP and FAP-N.* Conclusion*. Our study demonstrates that EoE-AP and EoE-D have different histology and outcomes. In addition, EoE-AP has clinical features similar to the FAP-N group.

## 1. Introduction

Eosinophilic esophagitis (EoE) in children and adolescents is associated with a variety of symptoms [1]. The consensus recommendation identified four dominant presenting symptoms of esophageal dysfunction: dysphagia (D), abdominal pain (AP), gastroesophageal reflux (GERD)/vomiting, and failure to thrive/feeding difficulty (FTT) [2]. Historically, GERD “evolved,” in its categorization as erosive esophagitis (EE), nonerosive gastroesophageal disease (NERD), and functional GERD [3, 4]. Clinicians know that the treatment for EE and NERD versus functional GERD is undoubtedly different [4, 5]. However, treatment of EoE is the same irrespective of the dominant symptom and has not been differentiated based on symptom.

Treatment of EoE in children includes steroids or diet. The symptom and/or histological improvement for these treatments vary from 50 to 100% [2] and prior studies did not often subgroup EoE patients based on the dominant presenting symptom. In practice, we observed that patients with EoE-AP have less favorable outcomes with treatment compared to EoE-D and that those children and adolescents with EoE-AP have clinical features similar to functional abdominal pain (FAP) except for the presence of eosinophils in the esophagus [2, 5]. Recent clinical trials on EoE include dysphagia as a required symptom and exclude patients who have abdominal pain without dysphagia [6]. To better define and characterize EoE, we divided the patients seen at our EoE clinic into four groups based on the dominant presenting symptom: dysphagia (EoE-D), abdominal pain (EoE-AP), GERD (EoE-GERD), and EoE-FTT.

We hypothesized that the two groups of EoE (EoE-D and EoE-AP) have different patterns of endoscopic and histologic findings and response to treatment. In addition, since EoE-AP has clinical features similar to FAP, we included a third group of patients, FAP with normal endoscopy and biopsy (FAP-N), for comparison [5]. Our study aims were to compare clinical features, endoscopic and histologic features, treatments, and outcomes of patients with EoE-AP to those with EoE-D and compare both groups to FAP-N. Our goal was to look for differentiating or comparable features between these groups and to see if EoE-AP has features similar to FAP.

## 2. Method

In this retrospective study, all children and adolescents with EoE seen over a period of 2.5 years (1/2010 to 6/2012) were divided into four groups based on the dominant presenting symptom.

Patients with EoE-AP had central or generalized abdominal pain. Diagnosis of EoE was confirmed as follows: esophageal biopsy with 15 or more eosinophils/HPF, no increased infiltration of eosinophils in the stomach or duodenum, preendoscopy treatment with proton pump inhibitors (omeprazole 40–60 mg or lansoprazole 30–60 mg), and/or a negative esophageal pH study (Bravo, Given Imaging, USA) [2, 7]. An additional cohort of children with FAP-N was selected from those seen in the clinic during the same time period with clinical criteria for FAP and having a normal EGD and biopsy [5]. Patients with celiac, Crohn's, drug allergy, and eosinophils in the stomach or duodenum were excluded. The following data were collected: symptoms, physical findings, allergic diseases, CBC, CMP, ESR, U/A, and endoscopic findings (furrows, white spots/exudates, concentric rings, and friability entered as absent (0) or present (1)). Three-four biopsies were obtained from duodenum, stomach, and distal and mid esophagus for histology. Severity of abdominal pain, nausea, vomiting, regurgitation, and heartburn was scored as follows: absent, 0; mild, 1 (does not interfere with daily activities); and severe, 2 (interferes with daily activities). A dysphagia score was assigned: absent, 0; mild, 1; severe, 2 [6, 8]; and an additional score for food impaction, 3, defined as impacted food requiring endoscopic removal [9]. Peak and mean esophageal eosinophil count at distal and mid esophagus were taken at diagnosis and follow-up [9, 10]. The GERD (presenting with heartburn or regurgitation without dysphagia) and FTT groups were not included because they were not part of our hypothesis for this study and were much smaller group.

Treatments included the following: topical steroids (fluticasone 880 mcg/day for age 1–10 years and 1760 mcg/day for 11–18 years, in four divided doses or budesonide 0.5 mg BID for patients up to 5 feet tall and 1 mg BID for those over 5 feet) and six food elimination or directed diet based on the allergy tests, [11, 12] based on a collaborative decision by the physician and family [12–14]. In some patients, a combination of diet and topical steroids was used. Clinical outcome was measured as follows: for dysphagia and abdominal pain as improved (score decreased by 1 or more), worsened (score increased by 1 or more), and no change (score same) and as composite score (calculated from the common five symptoms for each patient, given in Table 1, and then as a sum for all patients for each group), at diagnosis and follow-up [6, 8, 9]. EGD score and eosinophil count (peak and mean) were compared by comparing first endoscopy and the follow-up endoscopy [6, 9, 13, 14]. Statistical analysis was done using SPSS version 20. Significant *p* value was <0.05. For symptom outcome scores, a test of hypothesis was done by two-tailed binominal test. Institutional Review Board approval for this study was obtained through Advocate Health Care (Oak Brook, IL).

## 3. Data Analysis

Presenting symptoms and signs, associated allergic diseases, and treatments offered were entered into a two-step cluster analysis to determine whether there were distinct clusters [15]. These clusters were then cross-tabulated with the three groups of patients to determine whether the different groups of patients could be distinguished by clinical features. These are the following: age, abdominal pain, dysphagia, nausea, vomiting, regurgitation, anorexia, nocturnal awakening, early satiety, constipation, diarrhea, allergic rhinitis, eczema, asthma, PPI use, fluticasone, budesonide, and diet. In addition, a discriminant function analysis was performed to further explore clinical features associated with the three patient groups. Cross-tabulation with chi-square test was used to compare clinical outcomes across the three groups. Within each group, cross-tabulations and chi-square tests were used to examine the impact of treatment choices (oral steroid, directed diet, elemental diet, and other medications) on clinical outcomes [15].

## 4. Results

Patient characteristics by condition are shown in Table 1. Other than the dominant symptom, clinical features were similar in the two EoE groups. Nausea was seen more frequently in the EoE-AP and the clinical features of EoE-AP were similar to the FAP-N. Of the total 64 patients in EoE-D and 63 in EoE-AP group, 59 and 58, respectively, had PPI treatment and the remainder (10 patients out of study population of 127) had normal pH study. The mean WBC was 5320/mm^3^, 5420/mm^3^, and 4930/mm^3^ in the EoE-D, EoE-AP, and FAP groups. Eosinophil count more than 350/mm^3^ was 18.6%, 12.9%, and 6.2% in the three groups. ESR was done in 4/64 EoE-D patients and all patients with EoE-AP and FAP and was normal in all groups. EGD and biopsy findings are noted in Table 2. Visual EGD findings of the esophagus were more apparent in the EoE-D group, particularly the linear furrows and white exudates, and differences were statistically significant. When these two findings were combined, the differences increased and remained significant. Peak and mean eosinophil counts were as follows: EoE-D, 43.1 and 21.6; EoE-AP, 38.6 and 15.2 (*p*  0.06). Other histological features were not significant except for higher eosinophilic microabscess (*p*  0.001) in the EoE-D group.

## 5. Treatment and Outcomes

Treatments given are as follows: for EoE-D group, 45 (66.7%): topical steroids (43 fluticasone and 2 budesonide), six (9.3%): diet, nine (14.1%): topical steroids and diet, and two (3.1%): no treatment; for EoE-AP group, 37 (58.7%): topical steroids (35 fluticasone and 2 budesonide), two (3.2%): diet, 21 (33.3%): combination, and three (4.8%): no treatment. EoE-D group received more topical steroids while EoE-AP group received more of combined therapy with topical steroids and diet (chisq(9) = 18.4, *p* = 0.03). Follow-up period was up to 7.8 years (mean 3.4) in EoE-D, 8.8 years (mean 5.5) in EoE-AP, and in FAP 5.4 years (mean 4.8).

Table 3 shows that subjects with EoE-D had a significant improvement (86%) in their dominant symptom compared to EoE-AP (30%).  Table 4 shows similar results for composite symptom score. On the contrary, composite scores worsened in the EoE-AP group compared to EoE-D (*p* < 0.003). The test of hypothesis by the two-tailed binominal test for EoE-AP symptom outcome score showed worsening symptoms more often than 50% of the time compared to EoE-D and this was significant (*p* < 0.003); that is, the 69.8% not improved in symptoms in the EoE-AP group is higher than the 50% and significant. The FAP-N group improvement was similar to EoE-D group (Tables 3 and 4).

*EGD Findings*. 36/64 (56.3%) subjects with EoE-D and 28/63 (44.4%) with EoE-AP had follow-up endoscopy and biopsies with same protocol, done after 8–12 weeks of treatment (Table 5). Of the 36 subjects with EoE-D, 28/36 (77.8%) improved based on dysphagia, and the eosinophil count at diagnosis was 43.2/40.1 (peak/mean) and 25.8/22.7 at follow-up. This improvement was statistically significant and showed a linear correlation of eosinophils with the symptom improvement. For EoE-AP, 12/28 improved (42.8%) based on AP and the eosinophil count was 38.2/34.6 (peak/mean) and at follow-up 31.4/32.6 and did not have a linear correlation like EoE-D nor had a statistically significant difference in the eosinophils. In subjects who did not have follow-up endoscopy, 27/28 (96.4%) subjects with EoE-D and 7/35 (20%) of those with EoE-AP improved based on the dominant symptom.

**Figure 1:**
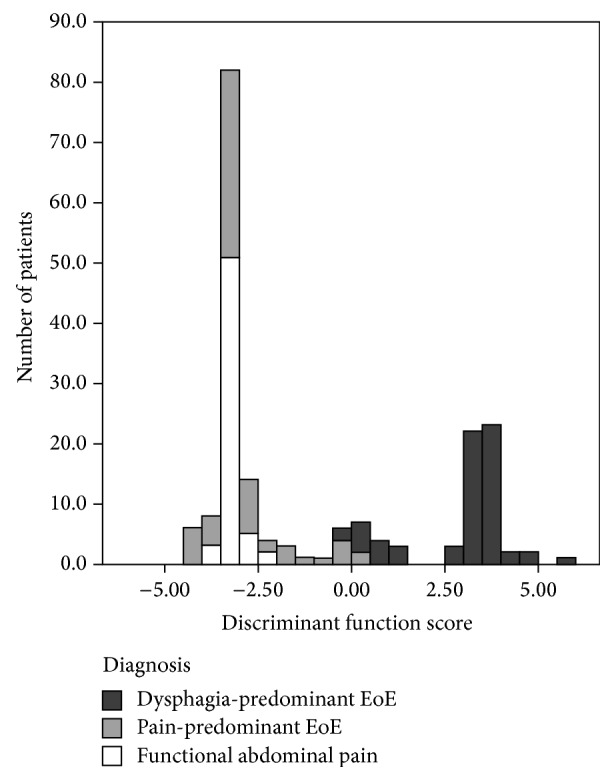


Figure 1 shows a histogram of the distribution of scores on the discriminant function with patient groups. EoE-AP and EoE-D subjects are perfectly discriminated, but EoE-AP subjects are both difficult to distinguish from FAP-N subjects (that is, they have similar features) and highly (though not perfectly) discriminable from EoE-D subjects.

Cluster analysis identified two clusters of patients with fair fit quality (average silhouette measure 0.4). Cluster 1 included all patients with EoE-AP and FAP and eight patients with EoE-D. Cluster 2 included the remaining 57 patients with EoE-D and no others. The most important predictors of cluster membership for EoE were the predominant presenting symptom, abdominal pain (Cluster 1) or dysphagia (Cluster 2). Food bolus was the next most important predictor; no patients in Cluster 1 had food bolus, while 37% of subjects in Cluster 2 had food bolus. Other symptoms were less predictive of cluster membership. In addition, Figure 1 shows the distribution of scores on the discriminant function with subject groups indicated by different shades. EoE-AP and EoE-D subjects are perfectly discriminated; that is, they are different groups. But EoE-AP subjects were difficult to distinguish from FAP patients (i.e., they have similar features) and highly (though not perfectly) discriminable from EoE-D patients. No specific treatments were associated with a greater rate of improvement overall or among groups.

## 6. Discussion

The diagnosis of EoE was established based on the presence of increased eosinophils in the esophagus, absence of eosinophils in the duodenum and stomach, and the presenting symptom. Thus eosinophilic gastroenteropathy was excluded and only a minority of patients had diarrhea. Our study raises the question whether EoE-AP and EoE-D are one disease with different features and outcomes or two different disease processes. To answer this question, we examined differentiating factors between the two groups including demographics, symptoms, visual endoscopic findings, and eosinophils in the esophagus. Of these variables, the features statistically significant were nausea in EoE-AP, visual endoscopic findings, especially when white exudates and furrows were combined, and eosinophilic microabscess for EoE-D.

With almost similar treatments, the EoE-D group showed a better and statistically significant improvement of the dominant symptom, while those with abdominal pain (EoE-AP) did not, and moreover the composite score in EoE-AP group worsened. Notably, in the EoE-D group (Table 5) there was a linear correlation with symptom and eosinophil improvement. Similar correlation was not seen with the EoE-AP. EoE-D group received topical steroids alone while the EoE-AP group was more likely to receive combined therapy with topical steroids and diet, and this may be because the EoE-AP group had more failures requiring additional therapy. Though the treatments were not controlled, individual treatments did not show an overall improvement among EoE groups, as shown on cluster analysis, which may be a surrogate of controlled treatment. The symptomatic improvement of EoE-D was similar to FAP while the clinical features of EoE-AP were similar to FAP except the eosinophils, and this raises the question whether EoE-AP and EoE-D are different or there is an overlap between EoE-AP and FAP.

According to the consensus recommendations, the diagnosis of EoE, in addition to eosinophils, should include symptoms of esophageal dysfunction [2]. While dysphagia is a cardinal symptom of esophageal dysfunction, abdominal pain does not hold a similar status as many other gastrointestinal diseases including FAP have abdominal pain as a primary symptom [16, 17]. Pentiuk et al. [18] looked at the correlation of symptom score and improvement in the eosinophils before and after treatment in 34 children and, in this group, about 70% had abdominal pain as the main symptom. Among 20 of these 34 children in histological remission, 17 (58%) continued to report symptoms and the majority of these patients had abdominal pain. In addition, there was no significant difference in the frequency and severity score of abdominal pain in patients in histological remission versus histologically active. The authors concluded that, despite improvement in the eosinophil count, a simultaneous symptom improvement failed to occur. A striking observation was that the majority of patients with this dissociation had abdominal pain as the dominant symptom [19]. Aceves et al. [8] found a direct correlation in improvement with dysphagia and early satiety/anorexia and eosinophil count, but there was not a similar correlation for other symptoms including abdominal pain. Another study by Assa'ad et al. [14] on 89 children with EoE showed a histological improvement on eosinophil numbers but the study did not mention the symptom follow-up. These studies note that EoE patients with abdominal pain as main symptom have less favorable outcome with treatment. Unlike children, adult patients present primarily with dysphagia and not abdominal pain. One explanation for this is that, in adults, EoE is more of a fibrostenotic disease, while in children it is often inflammatory. Additionally there is an understanding that the symptoms in children are “evolving,” and as they age dysphagia is seen in older children while abdominal pain is seen in younger children [2, 7], or it is possible that the esophageal inflammation in EoE-AP is an incidental finding.

Could there be another reason for the poor outcome in EoE-AP patients? Harris et al. [19] showed that a significant number of children and adolescents with EoE have psychosocial issues. The main symptoms of the 64 children in this EoE study were pain/physical discomfort (56%) or feeding/eating/appetite difficulties (45%); dysphagia was not mentioned as a symptom. About 30% of the patients had depression, 40% had anxiety, and about 60% had social issues. This study, unlike ours, did not group the patients by dysphagia or abdominal pain and suggested that EoE patients have psychosocial issues [19], which may explain the poor outcome in EoE-AP patients, which supports that there could be a functional component to the EoE-AP group.

A recent large prospective study by Butz et al. looked at the use of topical fluticasone versus placebo in a group of children and young adults with EoE [20]. Seventy percent of patients showed a complete remission of eosinophils at the end of three-month treatment compared to no subjects in placebo group. 69% of patients in the treatment group had abdominal pain and early satiety at the start of the study. Interestingly abdominal pain persisted in 61% of patients at the end of the study. This study clearly demonstrated that, in spite of histological improvement, there was no symptomatic improvement in the abdominal pain, further evidence that EoE-AP has a poor outcome.

Could there be other explanations for the EoE-AP patients to have a poor outcome? Talley et al. showed a positive correlation between functional gastrointestinal disease where the chief complaint is abdominal pain and associated duodenal eosinophilia [21]. Another study, related to this, but in children, showed similar correlation of dyspeptic symptoms and duodenal eosinophilia [22]. These studies suggest the possibility that eosinophils in the duodenum may be related to dyspeptic symptoms similar to FAP or visceral hyperalgesia. The biopsies of our patients did not have eosinophils in the duodenum or antrum, as the diagnosis will change to eosinophilic gastroenteropathy and this was an exclusion criteria as well. We also know that infection with resulting inflammation in the gut can lead to functional gastrointestinal symptoms or postinfectious functional dyspepsia (FD) or irritable bowel syndrome [23]. Various explanations are given for postinfectious FD-irritable bowel syndrome (IBS). The symptoms may depend on the type of infection and/or the site of infection, which affects the enteroendocrine cells and inflammatory cells like macrophages, eosinophils, and mast cells leading to altered motility. Can this explanation apply to EoE-AP patients? Eosinophil granules contain crystalloids composed of major basic protein, eosinophil-derived neurotoxin, eosinophil cation protein, and eosinophil peroxidase which release cytokines and chemokine, which alters smooth muscle function. In addition, eosinophil-derived products, leukotrienes, platelet-activating factor, and interleukin-13, can induce smooth muscle activation and proliferation resulting in inflammation, narrowing, or motility change [24, 25]. These in vitro studies speculate that a similar mechanism may apply to human beings. While it is possible that these mechanisms may apply to EoE-AP with esophageal eosinophil inflammation resulting in visceral hyperalgesia, it also questions whether a proximal esophageal inflammation would result in a distal dysmotility and abdominal pain.

In addition to the clinical features, we examined if the cluster analysis would differentiate the two groups [15]. Cluster analysis is a multidimensional scaling that seeks to group items (signs and symptoms) into a small number of clusters based on stronger associations between an item and the center of its cluster of items than between an item and the center of another cluster. The goodness of fit of the clusters is measured by the ability to interpret their membership as well as by the “silhouette” measure of the average distance from each item to its cluster center compared with the center of the next closest cluster. Silhouette scores range from −1 to 1, with scores above 0.2 being “fair” fit to the cluster structure and scores above 0.5 representing good fit to the cluster structure and our analysis showed that the silhouette scores for the two clusters were 0.4, closer to good fit. But cluster analysis only works with clinical features; it does not account for endoscopic results, the defining test for EoE. Both cluster analysis including “silhouette” measure and discriminant function grouped the EoE-AP and FAP-N as one group and different from EoE-D.

This is among the first studies to subgroup EoE in children and examine the clinical, endoscopic, and histological features and outcome of the two predominant groups of EoE and show that EoE-AP patients have poor outcomes and have features similar to FAP. Limitations of this study include the following: retrospective design, absence of validated symptom measures (though these scales were used in previously published studies) [8–11], not standardizing the dietary treatment, PPI use [26], and not correlating the exact treatment with outcomes. In addition, within the EoE-AP group, though the patients did not have overt dysphagia, it is possible, but less likely, that they may have adapted to gradual coping mechanisms and so dysphagia was not obvious.

In conclusion, because EoE is a clinicopathological condition, histology is but one feature and should not be considered diagnostic on its own. Our study presents evidence to suggest that EoE-AP and EoE-D have different outcomes and the EoE-AP group has features similar to FAP. We propose that, in the management of EoE-AP group of patients, if optimal diet or pharmacotherapy does not lead to symptomatic improvement or when there is dissociation between histological and symptomatic improvement, it is worth relooking not only at adherence to diet and/or medications but also at managing the EoE-AP group with methods successful in functional abdominal pain patients. This in fact may help better prognosticate the EoE-AP patients.

## Figures and Tables

**Table 1:** Clinical features of patients with eosinophilic esophagitis and functional abdominal pain.

	EoE-D *n* (%)	EoE-AP *n* (%)	FAP *n* (%)	EoE-D versus EoE-AP (*p*)	EoE-AP versus FAP (*p*)
Sex (%)					
Male	58 (90.6)	49 (77.8)	26 (42.6)	0.054	<0.001
Female	6 (9.4)	14 (22.2)	35 (57.4)		

Mean age, yr (range) at diagnosis	11.7 (3–17)	9.44 (2–17)	10.87 (4–17)		

Mean duration of FU, yr. (range)	3.4 (0.5–7.8)	5.5 (0.4–8.8)	4.8 (0.5–5.4)		

Presenting symptoms^a^, number (%)					
Dysphagia	64 (100)	1^b^ (1.6)	0 (0)	0.00	0.50
Abdominal pain^c^	8 (12.1)	63 (100)	61 (100)	0.00	0.07
Nausea	11 (16.7)	29 (45.3)	27 (44.3)	0.00	0.10
Vomiting	12 (18.2)	11 (17.2)	1 (1.6)	0.16	0.00
Regurgitation	5 (7.6)	7 (11.1)	3 (4.9)	0.19	0.12
Heartburn	7 (10.6)	6 (9.5)	1 (1.6)	0.21	0.05
Diarrhea	4 (6.25)	7 (11.1)	1 (1.6)	0.51	0.075

^a^Some patients had more than one presenting symptom.

^b^Patient initially presented with dysphagia; however subsequent visits showed abdominal pain as primary cause for distress.

^c^Abdominal pain was central or generalized.

**Table 2:** EGD and biopsy findings of patients.

	EoE-D	EoE-AP	*p*
EGD alterations, number (%)			
Linear furrows/vertical lines	53 (82.8)	33 (51.6)	0.008
White exudates	35 (54.7)	16 (25.4)	0.003
Linear furrows and white exudates	30 (46.9)	9 (14.3)	<0.001
Concentric rings/trachealization	6 (9.4)	3 (4.8)	0.16
Tears/crepe paper appearance	4 (6.3)	0	0.06

Eosinophil count peak/mean	43.1/21.6	38.6/15.2	0.06

Biopsy changes, number (%)			
Eosinophilic microabscesses	23 (35.9)	6 (9.5)	0.001
Basal epithelial hyperplasia	22 (34.4)	17 (27)	0.093
Papillomatosis	10 (15.6)	12 (19)	0.154
Spongiosis	9 (14.1)	10 (15.9)	0.176

FAP: visual changes and biopsies on EGD were normal.

**Table 3:** Symptom score change, dysphagia for EoE-D and abdominal pain for EoE-AP and FAP, baseline versus follow-up.

	EoE-D	EoE-AP	FAP	EoE-D versus EoE-AP (*p*)	EoE-AP versus FAP (*p*)
Total, number (%)	64	63	61		
Improved	55 (85.9)	19 (30.2)	49 (80.3)	<0.001	<0.001
Not improved	9 (14.1)	44 (69.8)	12 (19.7)		

EoE-D: baseline mean score = 1.5; sd = 0.69. Follow-up mean score = 0.6; sd = 0.53.

Mean difference is −0.89, which is significant (*p* < 0.001) by paired *t*-test.

EoE-AP group: baseline mean score = 1.2; sd = 0.43. Follow-up mean score = 1.4; sd = 0.77.

Mean difference is 0.20, which is not significant (*p* = 0.25) by paired *t*-test.

**Table 4:** Composite symptom score^*∗*^ change, baseline versus follow-up.

	EoE-D	EoE-AP	FAP
Patients	64	63	61
Baseline score	156	129	152
Follow-up score	47	135	79
	Composite score improved	Score worsened	Score improved

^*∗*^Symptoms included the following: dysphagia, abdominal pain, nausea, regurgitation, and vomiting.

EoE-D: baseline mean score = 2.4; sd = 0.92. Follow-up mean score = 0.7; sd = 0.67.

Mean difference is −1.68, which is significant (*p* < 0.001) by paired *t*-test.

EoE-AP: baseline mean score = 2.0; sd = 0.8. Follow-up mean score = 2.1; sd = 1.08.

Mean difference is 0.10, which is not significant (*p* = 0.57) by paired *t*-test.

**Table 5:** Follow-up EGD: correlation of symptom and eosinophil count changes.

	EoE-D baseline	^*∗*^On FU, improved	On FU, not improved	EoE-AP baseline	^*∗*^FU, improved	FU, not improved
FU EGD	36/64 patients	28 (77.8%)	8 (12.2%)	28/63 patients	12 (42.8%)	16 (57.2%)
Eos; peak/mean	43.6/40.8	25.2/22.8	64.6/56.6	38.5/34.6	31.2/30.4	59.5/49.5
NO FU EGD	28/64 patients	27 (96.4%)	1 (3.6%)	35/63 patients	7 (20%)	28 (80%)

^*∗*^FU: 36 (D) and 28 (AP) had follow-up EGD. Improvement based on single symptom score.

EoE-D group (*n* = 35): eosinophils baseline mean = 45.0; sd = 23.5. Follow-up mean = 17.7; sd = 20.3. Mean difference = −27.3; sd diff = 29.1, *p* < 0.001 by paired *t*.

EoE-AP group (*n* = 27): eosinophils baseline mean = 34.1; sd = 16.5. Follow-up mean = 32.0; sd = 32.0. Mean difference = −2.1; sd diff = 28, *p* = 0.7, not significant by paired *t*.

^*∗*^FU: follow-up.

## References

[1] Spergel J. M., Book W. M., Mays E. (2011). Variation in prevalence, diagnostic criteria, and initial management options for eosinophilic gastrointestinal diseases in the United States. *Journal of Pediatric Gastroenterology & Nutrition*.

[2] Liacouras C. A., Furuta G. T., Hirano I. (2011). Eosinophilic esophagitis: updated consensus recommendations for children and adults. *Journal of Allergy and Clinical Immunology*.

[3] Savarino E., Gemignani L., Pohl D. (2011). Oesophageal motility and bolus transit abnormalities increase in parallel with the severity of gastro-oesophageal reflux disease. *Alimentary Pharmacology and Therapeutics*.

[4] DeVault K. R., Achem S. R. (2000). Functional gastroesophageal reflux disease (GERD). *Current Treatment Options in Gastroenterology*.

[5] Schurman J. V., Friesen C. A., Danda C. E. (2005). Diagnosing functional abdominal pain with the Rome II criteria: parent, child, and clinician agreement. *Journal of Pediatric Gastroenterology and Nutrition*.

[6] Dohil R., Newbury R., Fox L., Bastian J., Aceves S. (2010). Oral viscous budesonide is effective in children with eosinophilic esophagitis in a randomized, placebo-controlled trial. *Gastroenterology*.

[7] Furuta G. T., Liacouras C. A., Collins M. H. (2007). Eosinophilic esophagitis in children and adults: a systematic review and consensus recommendations for diagnosis and treatment. *Gastroenterology*.

[8] Aceves S. S., Newbury R. O., Dohil M. A., Bastian J. F., Dohil R. (2009). A symptom scoring tool for identifying pediatric patients with eosinophilic esophagitis and correlating symptoms with inflammation. *Annals of Allergy, Asthma and Immunology*.

[9] Mackenzie S. H., Go M., Chadwick B. (2008). Eosinophilic oesophagitis in patients presenting with dysphagia—a prospective analysis. *Alimentary Pharmacology and Therapeutics*.

[10] Straumann A., Spichtin H.-P., Grize L., Bucher K. A., Beglinger C., Simon H.-U. (2003). Natural history of primary eosinophilic esophagitis: a follow-up of 30 adult patients for up to 11.5 years. *Gastroenterology*.

[11] Straumann A., Conus S., Degen L. (2010). Budesonide is effective in adolescent and adult patients with active eosinophilic esophagitis. *Gastroenterology*.

[12] Furuta G. T., Katzka D. A. (2015). Eosinophilic esophagitis. *The New England Journal of Medicine*.

[13] Liacouras C. A., Spergel J. M., Ruchelli E. (2005). Eosinophilic esophagitis: a 10-year experience in 381 children. *Clinical Gastroenterology and Hepatology*.

[14] Assa'ad A. H., Putnam P. E., Collins M. H. (2007). Pediatric patients with eosinophilic esophagitis: an 8-year follow-up. *The Journal of Allergy and Clinical Immunology*.

[15] Kaufman L. R., Rousseeuw P. (1990). *Finding Groups in Data: An Introduction Tocluster Analysis*.

[16] Mahajan L. A., Kaplan B., Wyllie R. W., Hyams J. S. (2006). Chronic abdominal pain of childhood and adolescence. *Pediatric Gastrointestinal and Liver Disease*.

[17] Monagas J. J., Hyman P. E., Sondheimer J. M. (2011). Dysphagia. *Pediatric Gastroenterology, Hepatology and Nutrition*.

[18] Pentiuk S., Putnam P. E., Collins M. H., Rothenberg M. E. (2009). Dissociation between symptoms and histological severity in pediatric eosinophilic esophagitis. *Journal of Pediatric Gastroenterology and Nutrition*.

[19] Harris R. F., Menard-Katcher C., Atkins D., Furuta G. T., Klinnert M. D. (2013). Psychosocial dysfunction in children and adolescents with eosinophilic esophagitis. *Journal of Pediatric Gastroenterology and Nutrition*.

[20] Butz B. K., Wen T., Gleich G. J. (2014). Efficacy, dose reduction, and resistance to high-dose fluticasone in patients with eosinophilic esophagitis. *Gastroenterology*.

[21] Talley N. J., Walker M. M., Aro P. (2007). Non-ulcer dyspepsia and duodenal eosinophilia: an adult endoscopic population-based case-control study. *Clinical Gastroenterology and Hepatology*.

[22] Friesen C. A., Kearns G. L., Andre L., Neustrom M., Roberts C. C., Abdel-Rahman S. M. (2004). Clinical efficacy and pharmacokinetics of montelukast in dyspeptic children with duodenal eosinophilia. *Journal of Pediatric Gastroenterology & Nutrition*.

[23] Futagami S., Itoh T., Sakamoto C. (2015). Systematic review with meta-analysis: post-infectious functional dyspepsia. *Alimentary Pharmacology and Therapeutics*.

[24] Rothenberg M. E., Cohen M. B. (2007). An eosinophil hypothesis for functional dyspepsia. *Clinical Gastroenterology and Hepatology*.

[25] Hogan S. P., Mishra A., Brandt E. B. (2001). A pathological function for eotaxin and eosinophils in eosinophilic gastrointestinal inflammation. *Nature Immunology*.

[26] Molina-Infante J., Ferrando-Lamana L., Ripoll C. (2011). Esophageal eosinophilic infiltration responds to proton pump inhibition in most adults. *Clinical Gastroenterology and Hepatology*.

